# Oxymatrine alleviates Echinococcus multilocularis infection by remodeling the liver immune microenvironment and intestinal flora homeostasis

**DOI:** 10.3389/fcimb.2025.1658336

**Published:** 2025-10-06

**Authors:** Yazhou Zhu, Peijiao Wu, Rou Wen, Jing Tang, Siyu Hou, Shiqin Yuan, Zihua Li, Ming Li, Wei Zhao

**Affiliations:** ^1^ School of Basic Medical Sciences, Ningxia Medical University, Yinchuan, China; ^2^ Ningxia Key Laboratory for Prevention of Common Infectious Diseases, Yinchuan,, China; ^3^ Department of Hepatobiliary Surgery, General Hospital of Ningxia Medical University, Yinchuan,, China; ^4^ Department of Medical Immunology and Pathogenic Biology, Ningxia Medical University, Yinchuan,, China

**Keywords:** OMT, Echinococcus multilocularis, 16S rRNA sequencing, liver immune microenvironment, alveolar echinococcosis

## Abstract

**Background:**

Alveolar echinococcosis (AE) is a zoonotic parasitic disease that poses a grave threat to human health. Recent studies have indicated that the gut microbiota plays a significant role in the pathogenesis of alveolar echinococcosis. Consequently, the quest for innovative microbial targeted modulators is anticipated to address the treatment of alveolar echinococcosis. Oxymatrine, an alkaloid extracted from the legume plant Sophora flavescens, has been demonstrated in research studies to regulate gut microbiota, thus treating various diseases, including rheumatoid arthritis and autoimmune encephalomyelitis. Nevertheless, the role of the aforementioned organism in alveolar echinococcosis remains to be elucidated.

**Methods:**

This study evaluated the effects of oxymatrine (OMT) at concentrations of 0.25 mM, 0.35 mM, 0.75 mM, 1 mM, and 1.25 mM on Echinococcus multilocularis protoscoleces *in vitro* over 48 hours, with cell viability assessed using the CCK-8 assay. Subsequently, an E. multilocularis infection model was established by intraperitoneal injection in mice. After three months of infection, the mice were treated daily with intraperitoneal injections of OMT at doses of 25 mg/kg, 50 mg/kg, or 100 mg/kg, alongside albendazole as a reference treatment, for two months. Fecal samples from each group were collected for 16S rRNA sequencing. Following treatment, tissue samples were analyzed. The liver and spleen indices were calculated by measuring mouse body weight, cyst weight, liver weight, and spleen weight. Hepatic pathological changes were examined using hematoxylin-eosin (H&E) and Masson’s trichrome staining. Additionally, flow cytometry was performed to quantify changes in hepatic CD4^+^ T cells, CD8^+^ T cells, and B cells.

**Results:**

*In vitro* experimental results demonstrated that treatment with oxymatrine at concentrations of 0.35 mM, 0.5 mM, 0.75 mM, 1.0 mM, and 1.25 mM significantly reduced the viability of *Echinococcus multilocularis* protoscoleces compared to the control group. The *in vivo* experimental results demonstrated that, compared with the control group, the 25 mg/kg, 50 mg/kg, and 100 mg/kg OMT-treated groups exhibited significantly reduced cyst weights, marked alleviation of liver inflammatory cell infiltration and fibrosis, and a significant increase in the number of hepatic CD8^+^ T cells. Furthermore, the 16S rRNA sequencing analysis revealed that OMT intervention enhanced the diversity of gut microbiota.

**Conclusion:**

Our data indicate that matrine can directly inhibit the growth of Echinococcus multilocularis *in vitro*, suggesting that matrine may play a therapeutic role in the early stage of alveolar echinococcosis. *In vivo* studies have shown that three months after infection, matrine can exert an anti-infection effect in the middle and late stages of alveolar echinococcosis by increasing the diversity of intestinal microbiota and the number of CD8^+^ T cells.

## 1 Introduction

Alveolar echinococcosis is a zoonotic parasitic disease caused by infection with Echinococcus multilocularis, which poses a serious threat to the health of patients and the development of livestock farming (Baykan et al., 2025; Beck et al., 2025). Alveolar echinococcosis occurs mainly in the liver, infiltrating and growing in the liver tissue, similar to the growth of malignant tumors, hence the name “worm cancer” (Xu and Ahan, 2020; Lundstrom-Stadelmann et al., 2025). Currently, the main treatment for alveolar echinococcosis in clinical practice is surgery combined with drug therapy (Autier et al., 2024). but neither of these treatments can cure patients in the late stages. Therefore, new treatments for multilocular echinococcosis are urgently needed.

A large number of studies have found that Chinese herbal medicine plays an important role in the treatment of infectious diseases. For example, *in vitro* anthelmintic efficacy of haloxylon salicornicum leaves extract using adult heamonchus contortus worms (Fatima, 2023), and antiparasitic activity of methanolic and ethyl acetate extracts of azadirachta indica against haemonchus contortus (Rehman et al., 2023). Oxymatrine (OMT) is the main active constituent of Sophora flavescens Aiton, a plant in the legume family (Jin et al., 2021; Li et al., 2023). Modern pharmacological studies have shown that OMT has multiple pharmacological effects, including anti-myocardial fibrosis, anti-inflammatory, anti-tumor, anti-arrhythmic and antiviral effects (Li et al., 2023). Compound Kushen Injection (CKI), a typical Traditional Chinese Medicine (TCM), has been clinically approved by the Chinese National Medical Products Administration (NMPA) for the treatment of cancer-related pain in China for over 20 years (Yang et al., 2021). In recent years, oxymatrine has emerged as a novel therapeutic approach for the management of various infectious diseases. The mice treated with OMT achieved better results in viscera index and survival rate than that of spiramycin. These results suggest that OMT are likely the sources of new drugs for toxoplasmosis (Zhang et al., 2016). In addition, oxymatrine can regulate the inflammatory factors TNF-α, NF-κB, and IL-6 through the TNF/NF-κB signaling pathway for the treatment of cryptosporidiosis (Zhang et al., 2024). However, the potential of oxymatrine as a novel therapeutic agent for alveolar echinococcosis remains to be elucidated.

In this study, an experimental intraperitoneal infection model was established through inoculation of Echinococcus multilocularis metacestodes. The therapeutic efficacy of oxymatrine in mice infected with E. multilocularis was detected and elucidated its modulation of hepatic immune cells and intestinal microbiota in these mice.

## 2 Materials and methods

### 2.1 Animal

The mice used in this study were 6–8 week old female C57BL/6J mice purchased from Jiangsu Jicui Yaokang Biotechnology Co (Production license number: SCXK (Su) 2023-0009). The mice were housed in an individually ventilated environment with a 12-hour light/12-hour dark cycle, and they were provided with food and water ad libitum. All experiments were conducted in accordance with the ethical standards set forth by the Ethics Committee of Ningxia Medical University (No.2024-G207).

### 2.2 Protoscolex separation

The methodology employed for the isolation of the protoscolex was as previously described. In briefly, The cyst tissue was extracted from the laboratory germ rat and divided into smaller fragments. The cut tissue was filtered through an 80-mesh filter and washed twice with PBS. It was then allowed to settle naturally for five minutes, after which the supernatant was discarded. The protoscole precipitate was digested with 1% trypsin for 30 minutes, washed twice with PBS, and allowed to settle naturally. Finally, 1% eosin staining was used to identify the activity of the protoscole.

### 2.3 Infection model construction and intervention

Following a one-week period of adaptive feeding, the protoscoleces (PSCs) were resuspended in phosphate buffered saline (PBS), and then 2000 protoscoleces were intraperitoneally injected into each C57BL/6N mouse. Three months following infection, the formation of lesions in the abdomen was identified via B-ultrasound in 20 mice. A total of 24 C57BL/6N mice were then randomly divided into four groups: an infection group, a 25 mg/kg oxymatrine group, a 50 mg/kg oxymatrine group, and a 100 mg/kg oxymatrine group. The mice in the infection group were administered 100 μL of phosphate-buffered saline (PBS, Hyclone) intraperitoneally on a daily basis. The mice in the 25 mg/kg oxymatrine group were intraperitoneally injected with oxymatrine solution (7.5 μL of a 10 mg/ml oxymatrine solution + 92.5 μL of PBS, SM4048-25mg, Beyotime, China) on a daily basis. The mice in the 50 mg/kg oxymatrine group were intraperitoneally injected with oxymatrine solution (15 μL of a 10 mg/ml oxymatrine solution + 85 μL of PBS) on a daily basis. The mice in the 100 mg/kg oxymatrine group were intraperitoneally injected with oxymatrine solution (30 μL of a 10 mg/ml oxymatrine solution + 70 μL of PBS) on a daily basis. All mice were administered the injection for a period of 30 consecutive days, after which samples were collected for analysis.

### 2.4 *In vitro* culture and intervention of protoscolece

The concentration of isolated multilocular Echinococcus was adjusted to 2000 PSCs/ml, and 100 μl was added to each well of a 96-well plate. A solution of 0.25 mM, 0.35mM, 0.5mM, 0.75mM, 1.0mM and 1.25mM oxymatrine was administered for a period of 48 hours. Five replicate wells were set up for each intervention group. Following the intervention, 10 μl of CCK-8 reagent was added to each well and incubated at 37°C for one hour. The absorbance was then detected at 450 nm using a microplate reader.

### 2.5 Detection of lactate dehydrogenase and alkaline phosphatase

LDH and ALT in the culture supernatant were measured using a lactate dehydrogenase assay kit (Beyotime Biotechnology, Shanghai, China) and an alkaline phosphatase assay kit(Solarbio, Beijing, China), strictly following the manufacturer’s instructions. Briefly, after the drug stimulation is completed, centrifuge the cell culture plates at 400 × g for 5 minutes using a multi-well plate centrifuge. Carefully aspirate the supernatant, add 150 μL of the LDH release reagent provided by the kit diluted 10 times with PBS (i.e., mix 1 volume of LDH release reagent with 10 volumes of PBS), gently shake the culture plates to ensure thorough mixing, and then place the plates back in the cell culture incubator for an additional 1-hour incubation. After the incubation, centrifuge at 400 × g for 5 minutes again. Subsequently, transfer 120 μL of the supernatant from each well to the corresponding wells of a new 96-well plate, and incubate at room temperature in the dark for 30 minutes. Measure the absorbance at 490 nm using a microplate reader. The formula for calculating cytotoxicity or cell death rate (%) is as follows: Cytotoxicity or cell death rate (%) = (Absorbance of treated sample - Absorbance of sample control well)/(Absorbance of maximum enzyme activity of cells - Absorbance of sample control well) × 100.

### 2.6 Fecal collection, DNA extraction and sequencing

Six fecal samples were collected from each group of mice and subjected to a DNA extraction process using a commercial DNA extraction kit(TIANGEN BIOTECH, Beijing, China), following the manufacturer’s instructions. In brief, The following reagents are required: 30 μL SDS (10%), 3 μL proteinase K (20 g/L), and 4 μL RNASe. These should be added to the pretreated 500 μL sample and mixed. The sample should then be stored in a 37°C water bath for 1 hour. Next, 100 μL of 5 M NaCl should be added to each tube, and the tubes should be inverted to mix. Then, 80 μL of a 10% CTAB/NaCl mixture (0.7 M NaCl and 10% CTAB) should be added, and the tubes should be gently mixed. Finally, the tubes should be placed in a 65°C water bath for 10 minutes. Following this, an equal volume of a phenol/chloroform/isoamyl alcohol (25:24:1) mixture should be added, and the contents thoroughly mixed. The tubes should then be placed in a centrifuge at 12,000 r/min for 10 minutes, after which the resultant pellet should be discarded. The tubes should then be filled with 0.6 vol. of isopropanol, and the contents gently mixed. The tubes should then be placed in a centrifuge at 12,000 g/min for 10 minutes, after which the resultant pellet should be discarded. The precipitate was then washed with 1 mL of pre-cooled 75% ethanol, followed by centrifugation at 7500 r/min for 5 min. The ethanol was then discarded, the precipitate dried slightly on a clean bench, and dissolved in 30 μL of TE buffer. Ten fecal samples were taken from each group for microbiome analysis.

To ensure the accuracy and reliability of sequencing data from the source, From DNA extraction to sequencing, strict sample testing and quality control were carried out, and each step strictly controls the sample quality to ensure the authenticity and reliability of the sequencing data. For the raw data (RawData) obtained after sequencing. First, the overlapping regions at both ends are used for splicing, and then low-quality sequences and chimeric sequences are filtered out to obtain high-quality CleanData. Next, based on CleanData. The sequences were denoised using QIIME2 D2DA2 to remove possible PCR amplification and sequencing errors in high-throughput sequencing data, thereby obtaining representative correct biological sequences ASV (amplicon sequence variants) and the abundance table of ASV. Then, further conduct subsequent α-diversity analysis, β-diversity analysis, species composition analysis and difference analysis, as well as functional composition and difference analysis.

### 2.7 Cyst inhibition rate and liver and spleen index detection

Following the administration, the mice were weighed. Anesthesia was induced in the mice with isoflurane, and the cyst tissues in the abdominal cavity, liver tissues, and spleen tissues were then separated and weighed. Cyst inhibition rate = (wet weight of cysts in the control group - wet weight of cysts in the experimental group)/wet weight of cysts in the control group × 100%. Liver or spleen index = (The weight of liver/spleen/the body weight of mouse) × 100.

### 2.8 HE staining and massion staining

It is imperative that the instructions for the reagents be followed precisely. The livers of the mice in each group were excised, fixed with 4% paraformaldehyde for 24 hours, embedded in paraffin, and cut into 6 μm slices to prepare tissue sections. The HE sections were prepared through a series of steps, beginning with dewaxing of the tissue samples. This was followed by dehydration with a range of alcohol gradients, staining with hematoxylin and eosin, and a subsequent transparentization process. Finally, the sections were sealed. They were then observed and photographed under a microscope.

### 2.9 The level of AST and ALT detecting

The automatic biochemical analyzer, Chemray 240, was employed to detect alterations in serum liver function indicators, namely alanine aminotransferase (ALT) and aspartate aminotransferase (AST) levels.

### 2.10 The immune cells in the liver and spleen were detected by flow cytometry

The lymphocytes from the spleen and liver were separated using the density gradient centrifugation method. In summary, the spleen and liver tissues were cut into small pieces of 1-2mm and placed on an 80 mesh filter for grinding. The resulting filtrate was collected and added to the upper layer of Percoll buffer, after which the mixture was subjected to centrifugation at 450g for 30 minutes. The lymphocyte layer was then carefully aspirated using a pipette. Finally, the lymphocytes were washed twice with PBS buffer and the cell count was performed. For flow cytometry, 1×10^6^ lymphocytes were collected into a centrifuge tube and subjected to cell staining. Subsequently, 2 μl of PE-CF594 anti-mouse CD3, APC-Cy7 anti-mouse CD4, PB anti-mouse CD8, FITC anti-mouse IFN-γ, and PE anti-mouse IL-4 antibodies were added at 4°C for 30 minutes. Subsequently, the cells were washed with PBS buffer and analyzed by flow cytometry.

### 2.11 Statistical analysis

The data from this study were analyzed using the statistical software packages SPSS 22.0 and GraphPad Prism 8.0. The normality of all data distributions was assessed using the Shapiro-Wilk test. Data that passed the normality test were analyzed using parametric tests (unpaired two-tailed Student’s t-test or one-way ANOVA). For data that violated the normality assumption, non-parametric tests (Mann-Whitney U test or Kruskal-Wallis test) were employed. The independent sample t-test or chi-square test were employed for the comparison of two groups, while the one-way analysis of variance or nonparametric test was used for the comparison of three or more groups. A p-value <0.05 was considered to be statistically significant.

## 3 Results

### 3.1 Oxymatrine inhibits the growth of protoscolex *in vitro*

**Figure 1:**
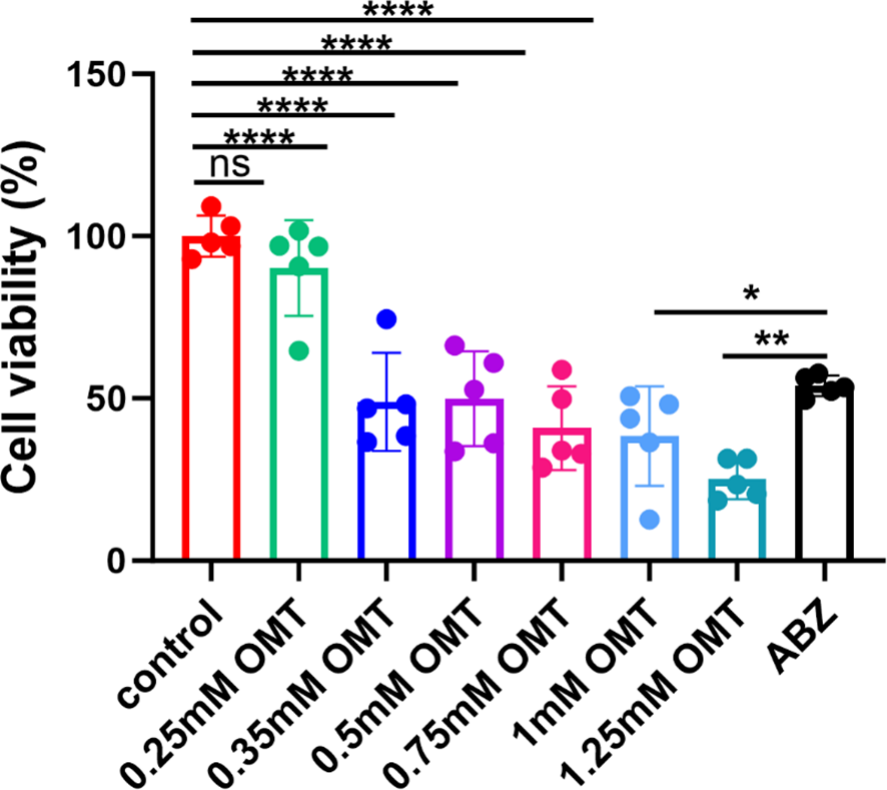
Oxymatrine inhibits the growth of protoscolex *in vitro*. Two hundred PSCs/100 μl were administered with 0 mM, 0.25 mM, 0.5 mM, 0.75 mM, 1.0 mM, and 1.25 mM oxymatrine for a period of 48 hours, and the viability of the protoscolex was assessed using a CCK8 assay. *P < 0.05, **P < 0.01, ****P < 0.001, ns indicates no statistical difference.

Protoscolex was administered with 0mM, 0.25mM, 0.5mM, 0.75mM, 1.0mM and 1.25mM oxymatrine for a period of 48h, the cell vitality was detected by CCK8 assay. Compared with control group, following the administration of 0.35 mM oxymatrine, a notable decline in the viability of the protoscolex was observed (*P* < 0.05). As the concentration of oxymatrine increases, there is a concomitant decrease in the vitality of the protoscolex. Albendazole is the drug of choice for the treatment of alveolar echinococcosis, and this study employed 0.5 μg/ml albendazole as a positive control. In comparison with the positive control group, the viability of protoscolex cells was found to be significantly reduced following the administration of oxymatrine at concentrations of 1.0 mM (*P* < 0.05)and 1.25 Mm (*P* < 0.01), as shown in **Figure 1**.

### 3.2 Oxymatrine alleviates liver tissue damage in mice infected with Echinococcus multilocularis

**Figure 2:**
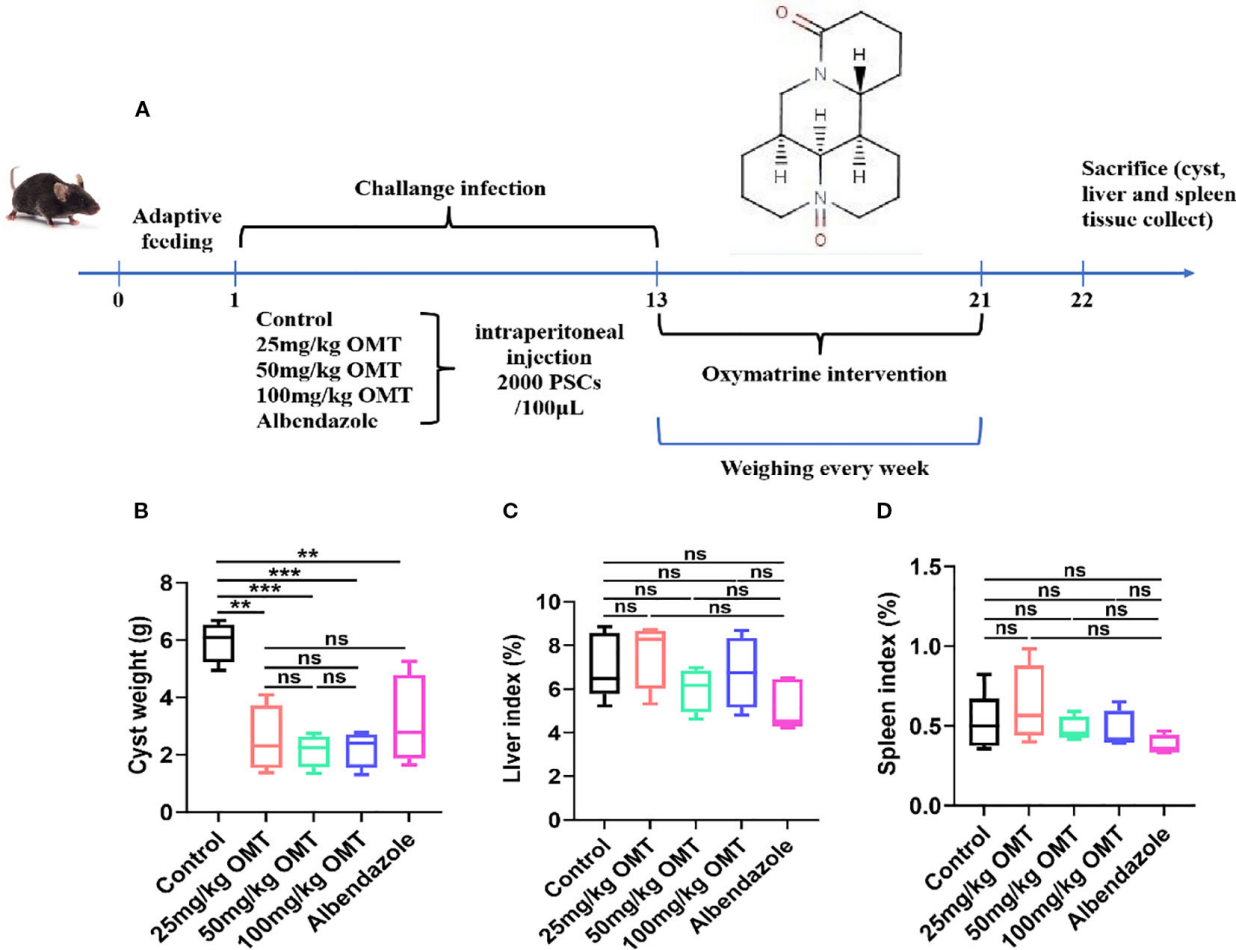
The inhibitory effects of oxymatrine on Echinococcus multilocularis infection in mice. **(A)** The schedule of Echinococcus multilocularis Infection and oxymatrine intervention. **(B)** The cyst weight of mouse. **(C)** Liver index of each group. Liver index(%) = The liver weight (g)/The body weight of mouse × 100. **(D)** Spleen index (%) = The spleen weight (g)/the body weight (g) × 100. **P < 0.01, ***P < 0.005, ns means no significance.

The construction of an infected mouse model was achieved by intraperitoneal injection of Echinococcus multilocularis. Intervention with oxymatrine was then conducted, after which samples were collected for testing. The specific timeline is shown in **Figure 2A**. The results showed that compared with the control group, after intervention with oxymatrine, the weight of cysts in mice was significantly reduced, while there were no significant changes in liver weight, spleen weight, liver index, and spleen index (**Figures 2B–D**). Albendazole is the preferred drug for the treatment of patients with alveolar echinococcosis in clinical practice. In this study, albendazole served as the positive control. Following oxymatrine intervention, no significant differences were observed in cyst weight, liver weight, and spleen weight within the abdominal cavity compared to the albendazole group (**Figures 2B–D**). These *in vivo* and *in vitro* results indicate that oxymatrine can inhibit the growth of multilocular echinococcosis.

### 3.3 Oxymatrine alleviates liver pathology in mice infected with Echinococcus multilocularis

**Figure 3:**
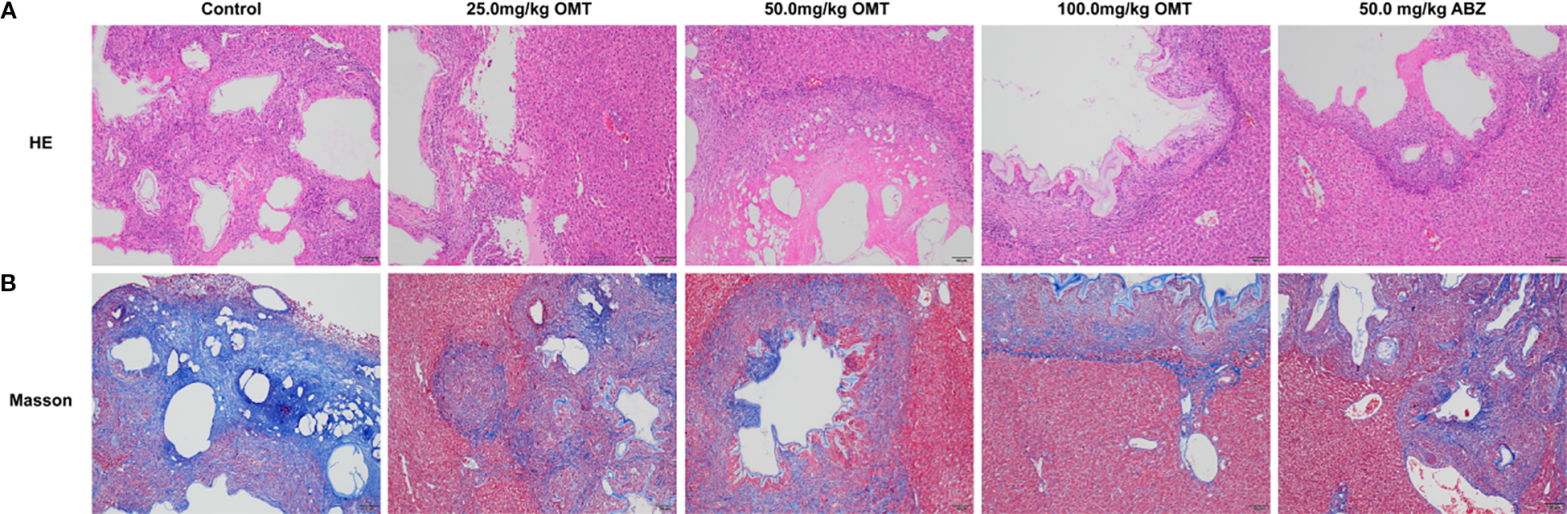
Oxymatrine alleviates liver pathology in mice infected with Echinococcus multilocularis. **(A)** HE staining was used to observe the pathological changes in the liver of mice in each group. **(B)** Massion staining was used to observe the pathological changes in the liver of mice in each group.

Furthermore, we investigated the impact of oxymatrine on mouse liver tissue pathology. The control group mice exhibited a significant presence of inflammatory cells near liver lesions, along with notable tissue fibrosis. Upon oxymatrine treatment, a decrease in inflammatory cell infiltration around liver tissue lesions and alleviation of liver tissue fibrosis were observed(**Figure 3**).

### 3.4 Oxymatrine regulate the balance of immune cells in the liver

**Figure 4:**
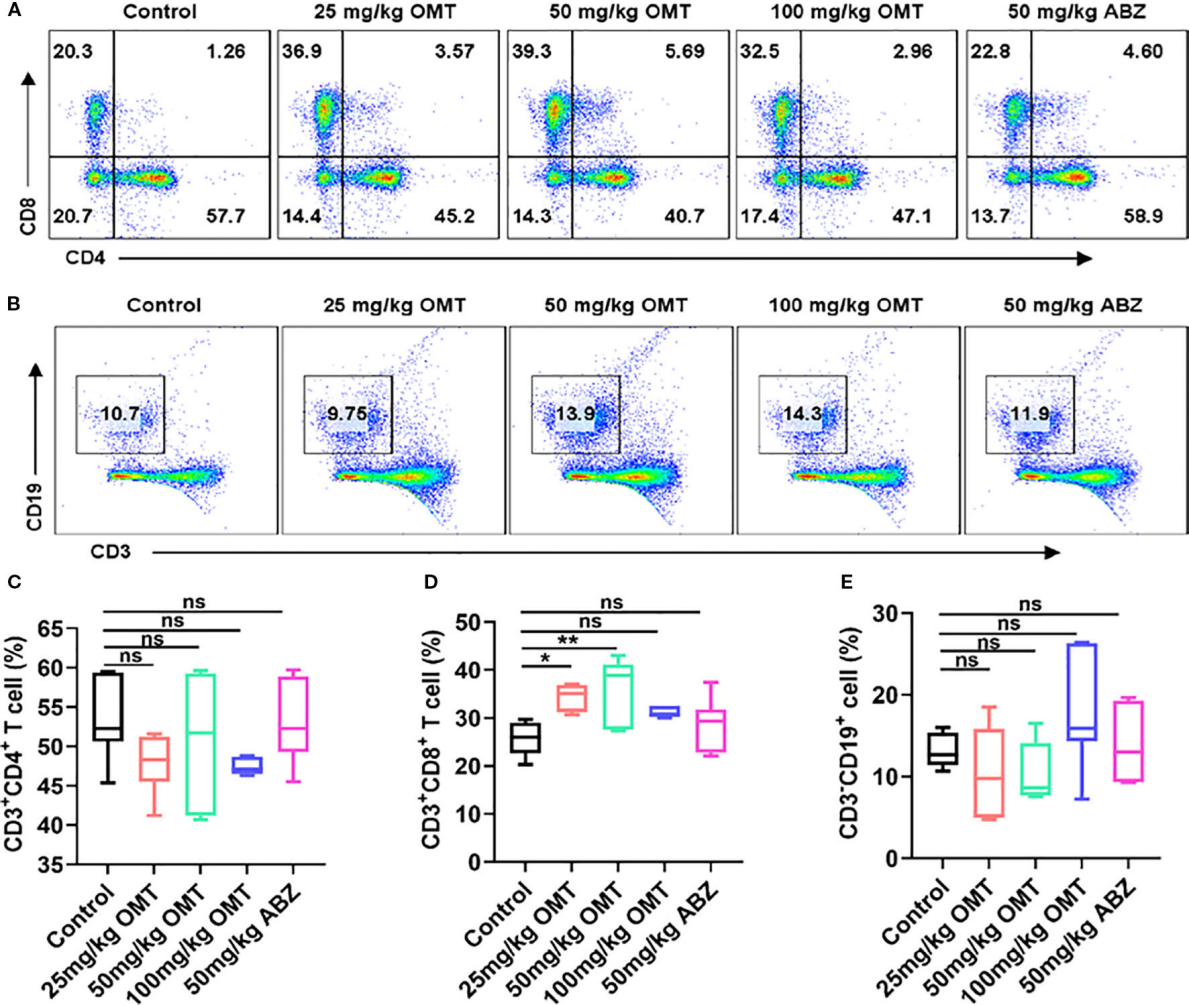
The proportion of CD4^+^ T cells, CD8^+^ T cells and B cells in hepatic tissue were detected by flow cytometry. **(A)** The proportion of CD4^+^ T cells and CD8^+^ T cells in hepatic tissue of each groups. **(B)** The proportion of B cells in hepatic tissue of each groups. **(C)** Statistical diagram of CD4^+^ T cell ratio in liver tissue of each group of mice. **(D)** Statistical diagram of CD8^+^ T cell ratio in liver tissue of each group of mice. **(E)** Statistical diagram of B cell ratio in liver tissue of each group of mice. *P < 0.05, **P < 0.01, ns means no significance.

To investigate the immunological mechanism by which oxymatrine mitigates Echinococcus multilocularis infection, liver mononuclear cells were isolated and analyzed using flow cytometry to monitor changes in CD4^+^ T cells, CD8^+^ T cells, and B cells. The findings revealed that compared to the control group, treatment with varying doses of oxymatrine (25mg/kg, 50mg/kg, and 100mg/kg) and albendazole did not result in significant alterations in CD4^+^ T cell (**Figures 4A, C**) and B cell populations within the liver of mice (**Figures 4B, E**). However, a notable increase was observed in the number of CD8^+^ T cells (**Figures 4A, D**). These results indicate that oxymatrine may combat multilocular echinococcosis infection by enhancing CD8^+^ T cell abundance in the liver.

### 3.6 Oxymatrine regulates gut microbial diversity in mice infected with Echinococcus multilocularis

**Figure 5:**
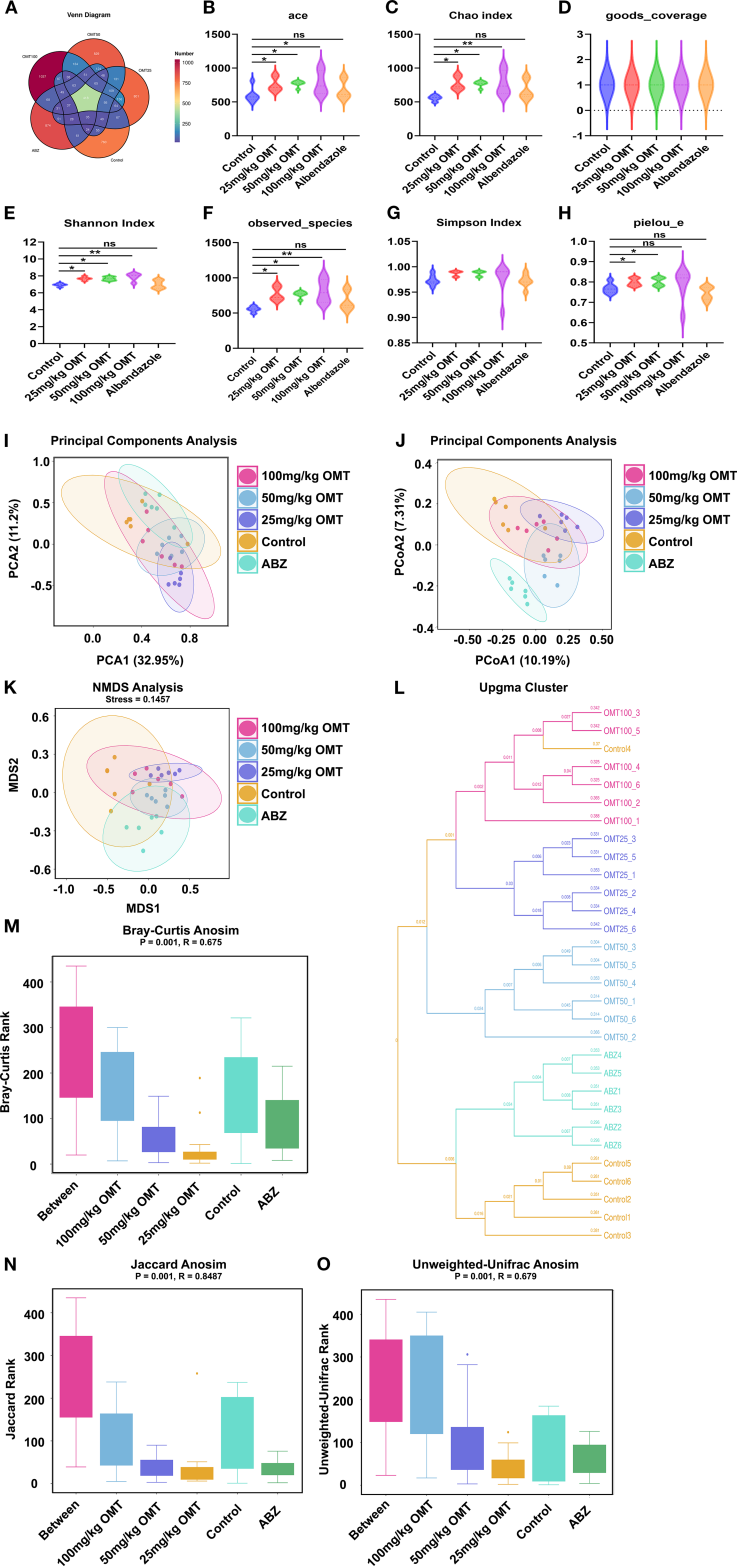
Gut microbiota alpha- and beta-diversities in each groups. **(A)** The Venn diagrams of intestinal microbiota in each group of mice. **(B–H)**. The effect of oxymatrine on the α diversity of intestinal microbiota. **(B)** Ace index. **(C)** Chao index. **(D)** good_coverage. **(E)** Shannon index. **(F)** Observed species. **(G)** Simpson index. **(H)** pielou_e. **(I–O)** The effect of oxymatrine on the β diversity of intestinal microbiota. **(I, J)**. Principle components analysis. **(K)** NMDS analysis. **(L)** Upgma cluster. **(M)**. Bray-curtis anosim. **(N)** Jaccard anosim. **(O)** Unweighted-unifrac anosim.

The Venn diagram in **Figure 5A** illustrates the distribution of species across different treatment groups: 760 species in the Control group, 801 species in the 25mg/kg OMT group, 829 species in the 50mg/kg OMT group, 1037 species in the 100mg/kg OMT group, and 874 species in the ABZ group were identified. Alpha diversity indices, such as Ace, Chao index, Good’s coverage, Shannon index, Simpson index, and Pielou’s evenness, are commonly utilized to assess community richness and diversity (**Figures 5B–H**). In this investigation, Ace, Chao, Shannon, and Pielou’s evenness values were significantly higher in the 25mg/kg OMT, 50mg/kg OMT, and 100mg/kg OMT groups compared to the Control group (P.05). Moreover, beta-diversity within the OMT group showed enhancements through Bray-Curtis analysis and Principal Coordinates Analysis (PCoA) utilizing the 16S data. The findings revealed substantial shifts in the gut microbiota of mice post-OMT intervention compared to the control group (**Figures 5I–K**). Additionally, UPGMA (Unweighted Pair Group Method with Arithmetic Mean) hierarchical clustering analysis indicated a high level of similarity in microbial community composition among sample groups (**Figure 5L**). Furthermore, Analysis of Similarities (Adonis) was employed to assess the similarity of each sample component, revealing significant distinctions between sample groups (**Figures 5M–O**).

### 3.7 OMT influenced the gut microbial abundance and phenotypes in mice infected with Echinococcus multilocularis

**Figure 6:**
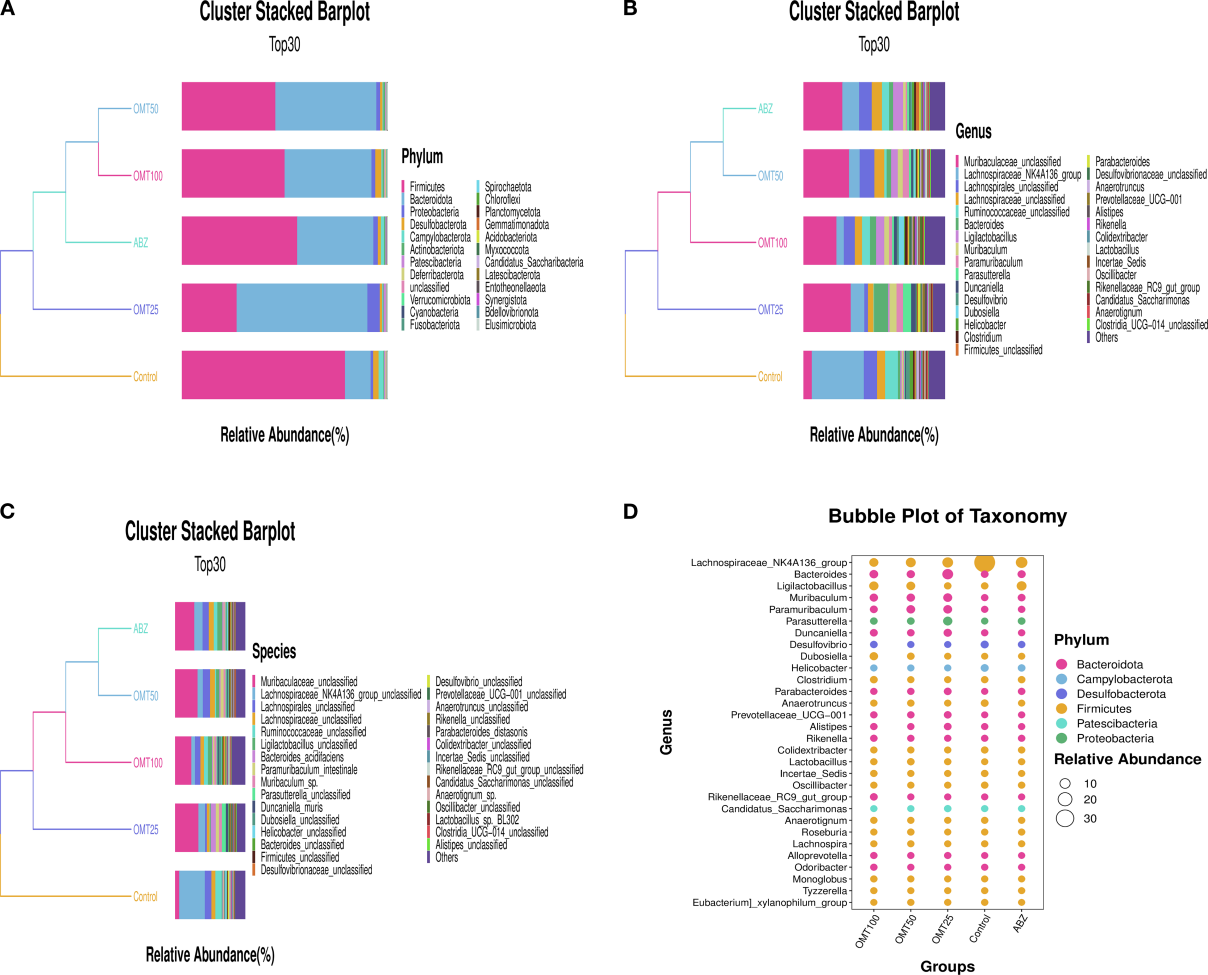
The effect of oxymatrine on the relative abundance of intestinal microbiota in mice. **(A)** The abundance of the top 30 microorganisms at the phylum level. **(B)** The abundance of the top 30 microorganisms at the genus level. **(C)** The abundance of the top 30 microorganisms at the species level. **(D)** Taxonomic bubble chart of differential microorganisms at the phylum level.

To investigate variations in gut microbiota among distinct mouse groups, the top 30 abundant microorganisms at the phylum, species, and genus levels were identified and annotated. Analysis at the phylum level revealed a significant decrease in Firmicutes microbiota following OMT intervention, accompanied by significant increases in Bacteroidota and Proteobacteria microbiota compared to the control group (**Figures 6A, D**). Similarly, at the genus level, A significant increase in Muribaculaceae and Bacteroides microbiota and a decrease in Lachnospiraceae microbiota were observed after OMT intervention compared to the control group. (**Figure 6B**). Additionally, species-level analysis indicated a significant decrease in Muribaculaceae post-OMT intervention compared to the control group (**Figure 6C**).

### 3.8 The difference of intestinal flora in mice of each group infected with Echinococcus multilocularis

**Figure 7:**
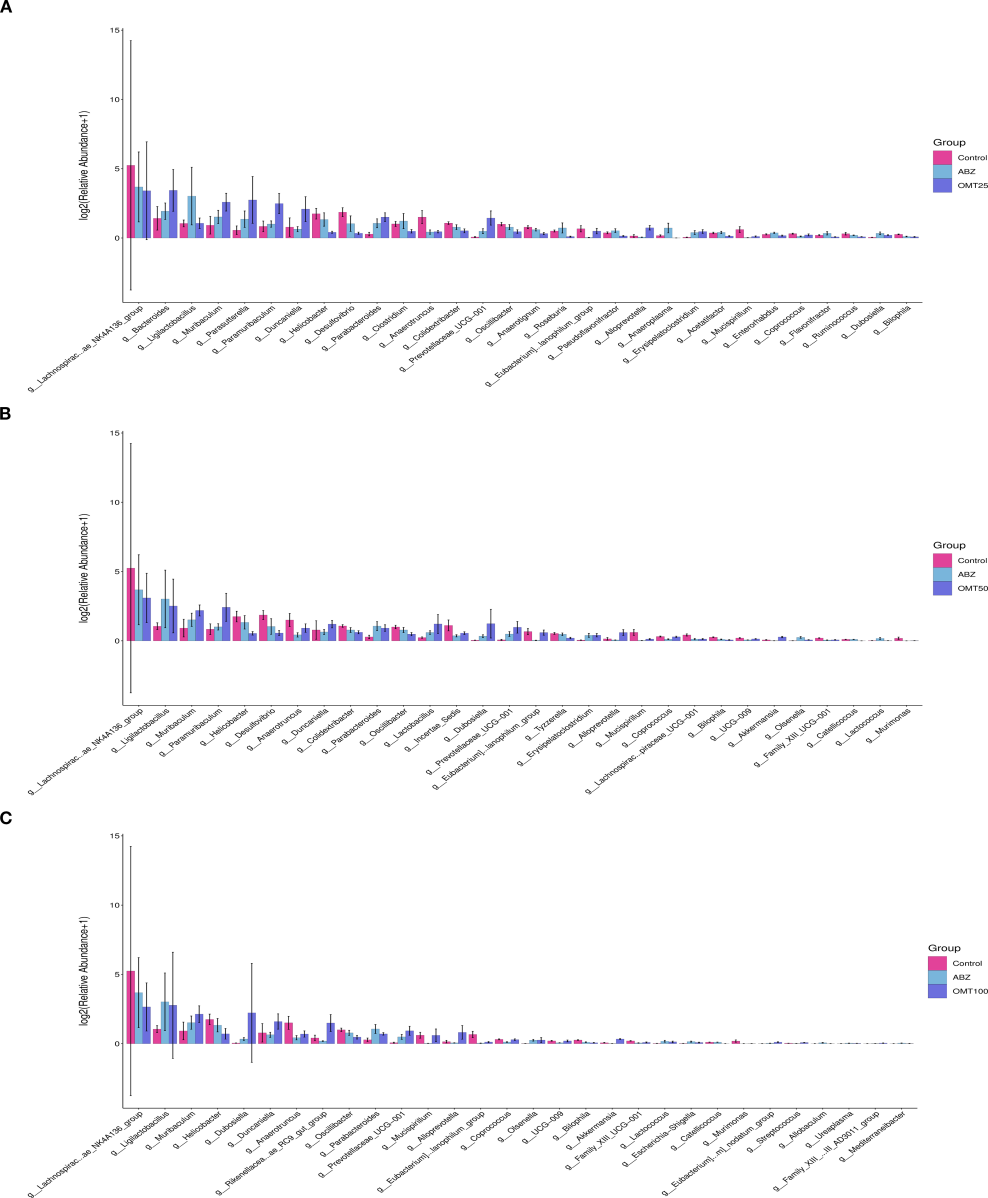
The influence of oxymatrine on the abundance of differential microorganisms. **(A)** The differential microorganisms between the control group, the ABZ group and the 25mg/kg OMT group. **(B)** The differential microorganisms between the control group, the ABZ group and the 50mg/kg OMT group. **(C)** The differential microorganisms between the control group, the ABZ group and the 100mg/kg OMT group.

To determine differences in gut microbiota among groups of mice after OMT intervention. The differences in the intestinal microbiota among groups were examined in detail, and the significance threshold for different species was set at *P* < 0.05. In total, 50 unique groups were identified at the genus level (**Supplementary Materials S1**). After conducting a differential analysis, thirty species showing significant differences were identified, and their abundances in different groups were presented in a histogram. Following treatment with 25mg/kg OMT, the groups g:Bacteroides, g:Muribaculum, g:Parabacteroides, and g:Paramibaculum exhibited significant up-regulation compared to the control group. Conversely, the groups g:Lachnospiraceae_NK4A136_group, g:Helicobacter, g:Desulfovibrio, g:Anaerotruncus, and g:Mucispirillum showed significant downregulation (**Figure 7A**). Following treatment with 50mg/kg OMT, the g_Ligilobacillus, g_Muribaculum, g_Parabacteroides, and g_Lactococcus groups exhibited significant upregulation compared to the control group. Conversely, the g:Lachnospiraceae_NK4A136_group, g:Helicobacter, and g:Desulfovibrio groups showed significant downregulation (**Figure 7B**). Following treatment with 100mg/kg OMT, the populations of g_Ligilobacillus, g_Muribaculum, g_Dubosiella, and g_Duncaniella in the mouse intestine exhibited significant upregulation compared to the control group. Conversely, the g:Lachnospiraceae_NK4A136_group, g:Helicobacter, and g:Anaerotruncus groups showed significant downregulation (**Figure 7C**).

## 4 Discussion

Alveolar echinococcosis poses a serious threat to human health. Due to the limitations of current treatments, developing alternative and complementary therapies, which particularly those with fewer side effects, better tolerance, lower costs, and improved quality of life outcomes, is essential. In recent years, alternative and complementary medicines have been widely studied for their role in controlling parasitic infections. For example, novel acaricidal activity of Vitex castus and Zingiber officinale extracts against the camel tick, Hyalomma dromedarii (Eltaly et al., 2022). In this study, oxymatrine as a potential complementary and alternative therapy for alveolar echinococcosis was explored.

Oxymatrine (OMT), a quinazoline alkaloid, has been extensively acknowledged for its diverse pharmacological activities, including anti-cancer, anti-diabetic, anti-viral, and anti-inflammatory effects, as well as its protective roles in the brain, liver, heart, lungs, blood vessels, gastrointestinal tract, bones, kidneys, and skin (Huan et al., 2023). In addition, oxymatrine has been shown to play a critical role in the management of infectious diseases. For example, Oxymatrine exhibits inhibitory effects on tachyzoite proliferation within the peritoneal cavity, thereby exerting anti-Toxoplasma activity (Zhang et al., 2016). Additionally, oxymatrine has demonstrated efficacy in the treatment of cryptosporidiosis through modulation of the NF-κB signaling pathway (Shi et al., 2024; Zhang et al., 2024). But whether oxymatrine has therapeutic effect on mice infected with multilocular echinococcosis is unclear. This study used Aslam et al.’s method to assess the dual effects of oxymatrine on organisms: beneficial and harmful (Aslam et al., 2023). In this study, in *in vitro* experiments, oxymatrine was directly applied to Echinococcus multilocularis. The results showed that the vitality of Echinococcus multilocularis decreased significantly. A similar finding was previously reported, Abubakar et al. demonstrated the *in vitro* anthelmintic activity of three plant extracts against various developmental stages of Haemonchus contortus (Fatima, 2023; Abubakar et al., 2024). Furthermore, an *in vivo* infection model was established via intraperitoneal injection of Echinococcus multilocularis larvae into mice, followed by daily administration of varying concentrations of oxymatrine via gavage to evaluate its therapeutic efficacy. The findings demonstrated a significant reduction in the weight of peritoneal lesions. Collectively, these results indicate that oxymatrine exhibits inhibitory effects on the growth of Echinococcus multilocularis both *in vitro* and *in vivo*. In addition, Parasitic infection can cause liver fibrosis and collagen accumulation (Winaya et al., 2022; Krishnaveni et al., 2023). In this study, it was found that after treatment with oxymatrine, liver fibrosis and collagen accumulation in mice were significantly reduced. Studies have shown that the reduction of fibrosis usually involves changes in the NF-κB and Nrf2/HO-1 signaling pathways (Ghaith et al., 2022). However, in this study, only the immunological mechanism of matrine in the treatment of alveolar echinococcosis was explored.

The gut microbiome is the largest and most complex microbiome in the human body, which plays an important role in the stability of the intestinal environment and the regulation of the host immune system (Sittipo et al., 2018) and the imbalance of intestinal microbiota structure and function are closely related to a variety of diseases (Zhang et al., 2022), including infectious diseases, such as viral hepatitis (Sehgal et al., 2020) and malaria (Sriboonvorakul et al., 2023). Therefore, targeting gut microbiota for disease treatment has emerged as a promising therapeutic approach. Research shows that oxymatrine alleviates NSAID-associated small bowel mucosal injury by regulating MIP-1/CCR1 signaling and gut microbiota (Chen et al., 2024). Several studies have demonstrated that oxymatrine can modulate the gut microbiota by regulating the PPARγ/COX-2 pathway, thereby mitigating pain and enhancing the integrity of the blood-brain barrier (BBB) compromised by bone cancer (Liu et al., 2024). However, there is currently no study reporting whether oxymatrine can alleviate Echinococcus multilocularis infection by modulating the gut microbiota. In this study, the effects of oxymatrine on intestinal microbiota in mice infected with Echinococcus multilocularis was investigated. The results showed that oxymatrine treatment could increase α diversity and β diversity of intestinal microbiota in mice, hinting that OMAT may play a therapeutic role by regulating the disorder of gut microflora in Echinococcus multilocularis-infected mice. Helminths cause chronic infections of over 1 billion people around the world (Specht and Keiser, 2023), creating a widespread acquired immunocompromised condition. Recent evidence from human and animal studies indicates that helminth infections, in particular of intestinal helminth, can influence the gut bacterial microbiota (Moles et al., 2022). In contrast, few studies have established a link between helminth infection and fungal compositions. In this study, the microbial composition of the gut of mice treated with oxymatrine was analyzed. The results showed that g:Bacteroides, g:Muribaculum, g:Parabacteroides, and g:Paramibaculum exhibited significant up-regulation compared to the control group. Conversely, the groups g:Lachnospiraceae_NK4A136_group, g:Helicobacter, g:Desulfovibrio, g:Anaerotruncus, and g:Mucispirillum showed significant downregulation. Bacteroides can ferment dietary fiber to produce short-chain fatty acids such as butyric acid, acetic acid and propionic acid, nourishing intestinal epithelial cells, anti-inflammation and maintaining intestinal health (Zafar and Saier, 2021). This also indicates that oxymatrine can inhibit the growth of Echinococcus multilocularis in mice by increasing the number of beneficial bacteria in the intestine.

In recent years, a large number of studies have shown that oxymatrine can act as an immunomodulator and exert anti-tumor effects by activating immune cells. Oxymatrine inhibits melanoma development by modulating the immune microenvironment and targeting the MYC/PD-L1 pathway (Li et al., 2023), and oxymatrine and Cisplatin Synergistically Enhance Anti-tumor Immunity of CD8^+^ T Cells in Non-small Cell Lung Cancer (Ye et al., 2018). Liver is the main organ of Echinococcus multilocularis parasitism, and liver immune cells are the main defense against liver pathogens (Beck et al., 2025). In this study, flow cytometry was used to detect changes in the number of immune cells in the liver. The results showed that the number of CD8^+^ T cells in the liver was significantly increased after oxymatrine intervention, which suggested that oxymatrine could induce an increase in the number of CD8^+^ T cells in the liver to resist pathogens.

## 5 Conclusion

Our data indicate that matrine can directly inhibit the growth of Echinococcus multilocularis *in vitro*, suggesting that matrine may play a therapeutic role in the early stage of alveolar echinococcosis. *In vivo* studies have shown that three months after infection, matrine can exert an anti-infection effect in the middle and late stages of alveolar echinococcosis by increasing the diversity of intestinal microbiota and the number of CD8^+^ T cells.

## Data Availability

The datasets presented in this study can be found in online repositories. The names of the repository/repositories and accession number(s) can be found in the article/**Supplementary Material**.
