# Supplementary material for: Oxymatrine alleviates Echinococcus multilocularis infection by remodeling the liver immune microenvironment and intestinal flora homeostasis
Source: Front Cell Infect Microbiol. 2025 Oct 6;15:1658336. doi: 10.3389/fcimb.2025.1658336 (PMC12535961; doi:10.3389/fcimb.2025.1658336)
Supplement: Supplementary file 1 [file Supplementaryfile1.doc]

| **Genus** | **log2FC** | **wilcox.test.p_value** | **q_value** | **significance** | **regulation** | **mean** |
| --- | --- | --- | --- | --- | --- | --- |
| g__Christensenellaceae_unclassified | Inf | 0.00 | 0.11 | yes | up | 0.01 |
| g__Lactococcus | -4.92 | 0.00 | 0.11 | yes | down | 0.07 |
| g__Akkermansia | 5.32 | 0.00 | 0.11 | yes | up | 0.08 |
| g__Streptococcus | 3.66 | 0.00 | 0.11 | yes | up | 0.05 |
| g__Bacteroides | 1.80 | 0.00 | 0.11 | yes | up | 6.32 |
| g__Paramuribaculum | 2.19 | 0.00 | 0.11 | yes | up | 2.81 |
| g__Helicobacter | -2.20 | 0.00 | 0.11 | yes | down | 0.93 |
| g__Alloprevotella | 4.05 | 0.00 | 0.11 | yes | up | 0.35 |
| g__Bacteroidales_unclassified | 2.86 | 0.00 | 0.11 | yes | up | 0.22 |
| g__Prevotella | 2.86 | 0.00 | 0.11 | yes | up | 0.04 |
| g__Allobaculum | -4.62 | 0.00 | 0.12 | yes | down | 0.02 |
| g__Ruminococcaceae_unclassified | -3.47 | 0.01 | 0.13 | yes | down | 2.78 |
| g__Ligilactobacillus | -2.71 | 0.01 | 0.13 | yes | down | 4.11 |
| g__Duncaniella | 2.54 | 0.01 | 0.13 | yes | up | 1.89 |
| g__Paraprevotella | -Inf | 0.01 | 0.14 | yes | down | 0.01 |
| g__Candidatus_Arthromitus | -5.25 | 0.01 | 0.16 | yes | down | 0.12 |
| g__Clostridia_vadinBB60_group_unclassified | -3.70 | 0.01 | 0.16 | yes | down | 0.42 |
| g__Sphingomonas | -1.66 | 0.01 | 0.16 | yes | down | 0.01 |
| g__Muribaculum | 1.43 | 0.01 | 0.16 | yes | up | 3.43 |
| g__Haemophilus | 3.54 | 0.01 | 0.18 | yes | up | 0.01 |
| g__Methyloversatilis | -3.60 | 0.01 | 0.18 | yes | down | 0.01 |
| g__Pseudoflavonifractor | -2.09 | 0.02 | 0.19 | yes | down | 0.27 |
| g__Ruminococcus | -1.23 | 0.02 | 0.19 | yes | down | 0.10 |
| g__Flavonifractor | -2.32 | 0.02 | 0.19 | yes | down | 0.16 |
| g__Tuzzerella | -1.80 | 0.02 | 0.19 | yes | down | 0.02 |
| g__Anaeroplasma | -7.76 | 0.02 | 0.20 | yes | down | 0.33 |
| g__Prevotella_7 | 2.01 | 0.02 | 0.20 | yes | up | 0.01 |
| g__Thalassospira | -Inf | 0.02 | 0.20 | yes | down | 0.01 |
| g__Tannerellaceae_unclassified | Inf | 0.02 | 0.20 | yes | up | 0.00 |
| g__Veillonella | Inf | 0.02 | 0.20 | yes | up | 0.00 |
| g__Caulobacter | -Inf | 0.02 | 0.20 | yes | down | 0.00 |
| g__Parasutterella | 1.87 | 0.03 | 0.20 | yes | up | 3.64 |
| g__Clostridium | -1.73 | 0.03 | 0.20 | yes | down | 0.86 |
| g__Enterorhabdus | -1.22 | 0.03 | 0.20 | yes | down | 0.21 |
| g__Acetatifactor | -1.70 | 0.03 | 0.20 | yes | down | 0.21 |
| g__UCG-005 | -3.77 | 0.03 | 0.22 | yes | down | 0.05 |
| g__Butyricicoccus | -2.72 | 0.04 | 0.25 | yes | down | 0.06 |
| g__Brevundimonas | -1.47 | 0.04 | 0.25 | yes | down | 0.01 |
| g__Lachnospirales_unclassified | -1.77 | 0.04 | 0.25 | yes | down | 5.80 |
| g__Prevotellaceae_UCG-001 | 2.08 | 0.04 | 0.25 | yes | up | 1.06 |
| g__Anaerotignum | -1.08 | 0.04 | 0.25 | yes | down | 0.38 |
| g__Eubacterium]_xylanophilum_group | 4.09 | 0.04 | 0.25 | yes | up | 0.22 |
| g__Megamonas | -1.07 | 0.04 | 0.25 | yes | down | 0.03 |
| g__Barnesiella | 2.81 | 0.04 | 0.27 | yes | up | 0.04 |
| g__Marvinbryantia | 3.14 | 0.05 | 0.32 | no | up | 0.15 |
| g__Anaerostipes | -Inf | 0.06 | 0.32 | no | down | 0.01 |
| g__RF39_unclassified | -Inf | 0.06 | 0.32 | no | down | 0.03 |
| g__Hydrogenophaga | -Inf | 0.06 | 0.32 | no | down | 0.00 |
| g__Mycoplasma | -Inf | 0.06 | 0.32 | no | down | 0.00 |
| g__Limnobacter | -Inf | 0.06 | 0.32 | no | down | 0.00 |
